# Role of cyanotoxins in the development and promotion of cancer

**DOI:** 10.1016/j.toxrep.2024.101798

**Published:** 2024-11-05

**Authors:** Siddharth Rajput, Shruti Jain, Debabrata Dash, Nidhi Gupta, Roshni Rajpoot, Chandrama Prakash Upadhyaya, Mohammed Latif Khan, Raj Kumar Koiri

**Affiliations:** a Biochemistry Laboratory, Department of Zoology, School of Biological Sciences, Dr. Harisingh Gour Vishwavidyalaya (A Central University), Sagar, Madhya Pradesh 470003, India; b Department of Biotechnology, School of Biological Sciences, Dr. Harisingh Gour Vishwavidyalaya (A Central University), Sagar, Madhya Pradesh 470003, India; c Department of Botany, School of Biological Sciences, Dr. Harisingh Gour Vishwavidyalaya (A Central University), Sagar, Madhya Pradesh 470003, India

**Keywords:** Cyanotoxins, Tumor, Carcinogenesis, Nodularin, Microcystin, Cylindrospermopsin

## Abstract

Cyanotoxins are primarily produced by different species of cyanobacteria, also known as blue-green algae, and have appeared to be environmental poisons that have various toxic effects on animal health, including humans. Cyanotoxins have been linked to the development and promotion of multiple cancers in recent studies. Important cyanotoxins, such as microcystins, nodularins, and cylindrospermopsin, have been found to play significant roles in developing and promoting various cancers. These toxins are generally responsible for oxidative stress, DNA damage, and disrupt cellular signaling pathways thus the development of cancers in various cells. Cancer is a multistep process caused by multiple mutations in normal cells. Microcystin-LR inhibits protein phosphatases (PP1 and PP2A), which leads to abnormal cell proliferation and tumor development. Similar inhibition of PP1 and PP2A is shown by nodularin, and in fact, their mechanism of carcinogenesis is the same as that of microcystins to **s**ome extent. Cylindrospermopsin inhibits protein synthesis and thus has genotoxic effects and may promote the development of cancer. Anatoxin-a and saxitoxins are well-known neurotoxins but, are thought to have indirect carcinogenic effects based on the fact that they can induce oxidative stress and DNA damage in cells by producing reactive oxygen species, thus further studies are needed to fully elucidate their role in the development and promotion of cancer. This review provides a detailed account of how different cyanotoxins play a role in the development and promotion of cancer.

## Highlights

• Cyanotoxins cause hepatotoxicity, genotoxicity, and neurotoxicity based on their target organs and mechanism of action.
• Cyanotoxins promote carcinogenesis by regulating different cellular pathways.
• Cyanotoxin promotes the formation of ROS and causes DNA damage within the cells, which is responsible for cancer development.

## Graphical Abstract

**Figure:**
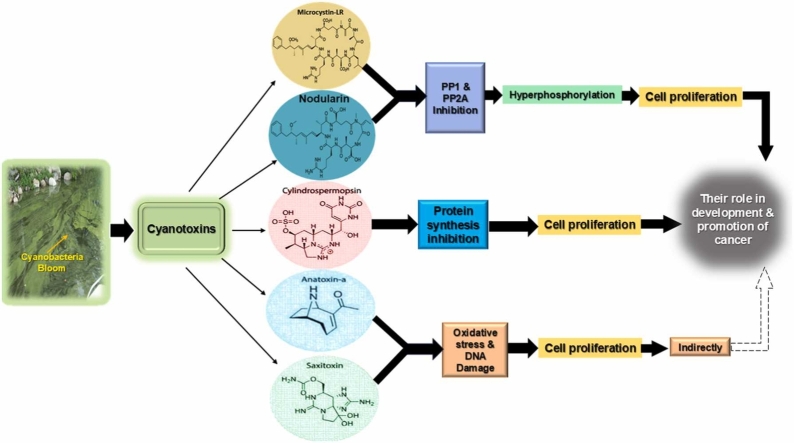

## 1 Introduction

A class of poisonous substances known as cyanotoxins is toxic to all animals, including humans. Blue-green algae, or cyanobacteria, are the producers of cyanotoxins, which are secondary metabolites. The International Agency for Research on Cancer (IARC) has classified certain cyanotoxins, such as microcystin-LR (MC-LR), as possible human carcinogens [1]. Fresh, brackish, and marine water are common homes to them [2]. In freshwater and marine environments, cyanobacterial blooms are mostly caused by eutrophication and climate change. Key drivers of cyanobacterial blooms are eutrophication and warming. Warming promotes cyanobacterial abundance in a natural plankton community and that eutrophication enhances cyanobacterial biomass [3]. Increasing proliferation of cyanobacterial blooms is likely a potential threat to the environment [4]. An increase in cyanobacterial growth and cyanotoxin production poses a concern to human and animal health. Cyanotoxin can result in life-threatening chronic illnesses such as cancer and severe poisoning. Thus, from the point of view of ecotoxicology and toxicology, cyanotoxins play an important role in environmental chemistry [5].

Exposure of cyanotoxins to humans and other animals can occur in two main ways: directly or indirectly. Direct exposure occurs by drinking contaminated water or coming into contact with it during swimming or bathing, breathing in tiny particles during activities like showering or water sports, or even through medical procedures. Indirect exposure occurs by consumption of animal or plant products that have been affected by cyanotoxins [6], [7], [8]. Additionally, research shows that these toxins can bioaccumulate in the food chain, strengthening their harmful effects over time [6]. The aerosolization of toxic cyanobacteria and cyanotoxins is also a critical concern as an emerging exposure route for potential risks to environmental and human health. Toxic cyanobacteria and their cyanotoxins can be aerosolized by a bubble-bursting process associated with a wind-driven wave mechanism [9]. Besides these, the primary concern of human exposure to cyanotoxins arises from the consumption of contaminated drinking water, which has been linked to severe poisoning incidents and even mortalities worldwide [9]. These toxins are known to act rapidly, often within minutes of exposure, and they can be fatal to animals in many cases. Their toxic effects are generally not visible till an animal has ingested a dose of toxin close to lethal levels, making early detection difficult [6]. A recent study, investigated the effects of perinatal exposure to the cyanobacterial toxin β-N-methylamino-L-alanine (BMAA) on neurodevelopment [10].

In a recent study across 31 countries revealed that cyanotoxins are widely present in various food sources worldwide. Notably, 68 % of the research found microcystin levels exceeding safe limits for daily intake. Cyanotoxins were also commonly detected in fish, with herbivorous species showing the highest concentrations, though omnivores like *Oreochromis niloticus* had a greater tendency to accumulate these toxins. Besides fish, crustaceans and bivalves also accumulated significant levels of cyanotoxins [11]. Additionally, over half (57 %) of cyanobacteria-based food supplements, particularly those containing *Aphanizomenon flos-aquae*, contained cyanotoxins, which raises concerns about product safety [11]**.** Another study revealed that due to different climatic and environmental factors, the levels of cyanotoxin vary significantly in freshwater bodies across various European lakes and reservoir [12]. In Italy, Microcystin (MC) levels rise from 3.4 µg/L in 2009–5.205 µg/L in 2010, with notable peaks in May, October, February, and November at Lake Vico. The highest recorded MC concentration reached 100 µg/L in Lake Alto Flumendosa during the same months. Spain reported MC levels of 18.6 µg/L in Reservoir Rosarito, alongside anatoxins (ANA) at 2.1 µg/L and saxitoxins (STXs) at 0.12 µg/L, peaking in September for MCs and ANA and July for STXs, while Ojos Reservoir exhibited lower MC concentrations of 0.17 µg/L during spring and summer. In Portugal, peaks varied across reservoirs, with MCs reaching 2.58 µg/L in Alvito and 7.2 µg/L in Roxo. Greece recorded MCs peaking at 19 µg/L and STXs at 2.1 µg/L in Lake Pamvotis, with peaks occurring in March and September, while Lake Marathonas showed concentrations of 0.717 µg/L in February and September-October. Turkey reported the highest MC concentration at 20.5 µg/L in Lake Egirdir, peaking in April and August. Poland saw significant MC levels of 30.68 µg/L in Mytycze and 23.62 µg/L in Tomaszne, peaking from mid-August to September. In Germany, cylindrospermopsin (CYN) reached 1.8 µg/L in Lakes Langer See and Melangsee, while MCs peaked at 6.7 µg/L in Lake Klostersee in October. Lastly, Russia reported MC concentrations reaching 41.37 µg/L and ANA levels at 0.54 µg/L in Lakes Suzdal and Sestroretskij Razliv, with peaks between August and September. [12]. A study revealed that in the water of Sagar Lake (Lakha Banjara Lake) India, Microcystin-LR was found at a concentration of 0.67 µg/mL, which is far beyond the guideline set by WHO [1].

A comprehensive study documented the identification of 1118 cyanotoxins across 869 freshwater ecosystems in 66 countries [13]. In their study, microcystins were the most frequently detected cyanotoxins, making up 63 % (699 of 1118) of the cases, followed by cylindrospermopsin (10 %; 107 cases), anatoxins (9 %; 100 cases), and saxitoxins (8 %; 93 cases). Nodularins were the least frequently reported, accounting for just 2 % (19 cases). Additionally, in 9 % of cases (100 of 1118), cyanotoxins were either not analysed or not specified. Moreover, there were 183 cases of cyanotoxin-related poisoning involving humans and animals. Geographically, North and Central America had the highest percentage of poisonings (39 % cases), followed by Europe (20 % cases), Australia and New Zealand (15 % cases), and Africa (11 % cases). The lowest percentages were reported in Asia (8 % cases) and South America (8 % cases). Remarkably, 63 % of poisoning incidents involved animals, while 32 % affected only humans [13]. A study revealed that in the Arabian Gulf, desalinated water stored often contains cyanobacteria. Study revealed that 80 % of urban and 33 % of rural impoundments are affected with cyanobacteria and cyanotoxins. Neurotoxins like β-N-Methylamino-L-alanine (BMAA) were absent, but a toxic BMAA isomer N-(2-aminoethyl) glycine (AEG) was found in 91.7 % of rural samples. Microcystin-LR, a hepatotoxin, exceeded WHO limits in 30 % of urban water tanks, highlighting storage risks [14]. In a similar study Cyanotoxins were also found in 95 waterbodies across 15 African countries, excluding the central region. Microcystins were the most prevalent reported in 98.9 % of cases, while other toxins like anatoxin-a (5.3 %), cylindrospermopsin (2.1 %), nodularins (2.1 %), homoanatoxin-a (1.1 %), and β-N-methylamino-l-alanine (1.1 %) were rare. The highest levels of microcystins and nodularins were recorded in South African lakes Nhlanganzwani (49,410 μg/L) and Zeekoevlei (347,000 μg/g). The 63 % of the waterbodies exceeded the WHO guideline for lifetime drinking water safety (1 μg/L), highlighting the widespread risk of cyanotoxin contamination [15].

Research that has been published indicates that of all human diseases, cancer has the greatest clinical, social, and financial burden regarding cause-specific disability-adjusted life years (DALYs). According to estimates, ischemic heart disease is the leading cause of mortality globally (8.97 million deaths), with cancer predicted to overtake it in 2060 (∼18.63 million fatalities). Lung, liver, and stomach cancers are the three most lethal cancers in the general population, while lung and breast cancers are the main causes of cancer-related mortality in men and women, respectively [16].

Previously published studies are primarily focused on individual cyanotoxins and their toxicological effects such as hepatotoxicity, neurotoxicity, and dermatotoxicity. Instead, this is a comprehensive study on the role of different cyanotoxins in the development and promotion of cancer. In our study, we comprehensibly discussed different types of cyanotoxins, their toxicity, and the carcinogenic mechanisms associated with them, such as oxidative stress, DNA damage, and modulation of some gene expression, such as tumor necrosis factor-α (TNF-α). This is an interdisciplinary study integrating molecular biology and toxicology. Additionally, the study highlights mitigation strategies for cyanotoxins along with health protection approaches.

## 2 Overview of cyanotoxins: Types, target organs and toxic effects

Cyanotoxins can be classified into five categories based on the organ they target and induce toxicity: dermatotoxins, cytotoxins, neurotoxins, hepatotoxins, and irritating toxins [17]. Lipidic, heterocyclic, and peptide compounds are the three categories into which cyanotoxins can be divided based on their chemical structures. Cyanobacterial lipopolysaccharides, an essential part of the cell wall of all cyanobacteria, are also categorized as cyanotoxins due to their harmful properties [5]. The table below provides a comprehensive overview of various cyanotoxin types, target organs, toxic effects, LD50, and producing species (Table 1).

**Table 1:** Overview of Cyanotoxins: Types, Target Organs, Toxic Effects, LD50, and Producing Genera.

**Cyanotoxins**	**Target Organs**	**Description**	**Toxic Effects**	**LD**_**50**_	**Producing Genera**	**References**
Microcystin (MCs) (Hepatotoxins)	Liver and also affects kidney, reproductive tissue, colon, and brain.	Cyclic heptapeptides, MC-LR, were categorized as a group 2B carcinogen by the International Agency for Research on Cancer in 2010. Microcystin-LR (MC-LR) acts as a tumor initiator, WHO recommends a maximum concentration of 1 µg/L for microcystin-LR in drinking water.	Hepatotoxic, Inhibition of eukaryotic protein phosphatases (PP1 and PP2A).	MC-LR 50 μg/kg b.w. ip in mice. MC-LA 50 μg/kg b.w ip in mice. MC-YR 70μg/kg b.w ip in mice. MC-RR 300-600 μg/kg b.w ip in mice.	*Microcystis* *Planktothrix* *Anabaena* *Nostoc* *Aphanizomenon*	[13], [18], [19], [20], [21], [22], [23], [24]
Nodularin (NODs) (Hepatotoxins)	Liver	Cyclic pentapeptides, hepatotoxic, tumor promoters, & carcinogenic. Nodularin-R was the first identified type.	Inhibition of protein phosphatases (PP1, PP2A and PP3), liver haemorrhage.	50 μg/kg b.w. ip in mice.	*Nodularia* *Nostoc*	[20], [25], [26], [27]
Cylindro-spermopsin (CYNs) (Hepatotoxins)	Liver, nervous system. heart, thymus, and kidney.	Guanidine alkaloids, hepatotoxic, cytotoxic, neurotoxic, genotoxic, may promote initiation and progression of tumors.	Irreversible inhibitor of protein biosynthesis, inhibition of glutathione and cytochrome P450.	2100 μg /kg ip in mice. 4400–6900 μg/kg b.w. orally in mice.	*Raphidiopsis* *Anabaena* *Aphanizomenon* *Chrysosporum* *Oscillatoria* *Umezakia*	[13], [20], [28], [29], [30]
Anatoxin-a (ATXs) (Neurotoxins)	Brain, respiratory system, muscles, and cardio-vascular system.	Neurotoxic, bicyclic secondary amine, an inhibitor of the enzyme acetylcholinesterase (AChE), promote muscular paralysis, reactive oxygen species (ROS) production, and mitochondrial dysfunction. very fast death factor (VFDF).	Neuro-muscular blocking agent, death by asphyxiation, persistent stimulation. Pre and postsynaptic effects, mimics neurotransmitter acetylcholine and blocks acetylcholine-esterase.	375 µg/kg b.w. ip in mice. 100 µg/kg b.w iv in mice.	*Anabaena* *Aphanizomenon* *Raphidiopsis* *Oscillatoria* *Planktothrix* *Phormidium* *Tychonema* *Lyngbya*	[5], [21], [31], [32], [33], [34], [35], [36]
Anatoxin-a(S) (Neurotoxins)	Muscles	Potent neurotoxic, guanidine methyl phosphate ester, water-soluble, highly toxic. promote hyper salivation, diarrhoea, paralysis, and asphyxiation.	Irreversibly blocks Acetylcholine-esterase (AChE), and affects signal transduction in neurons. Nerve hyperexcitability.	20-40 μg/kg b.w. ip in mice and rats.	*Anabaena* *lemmermanni*	[13], [19]
Homoanatoxin-a (Neurotoxins)	Muscles	Alkaloids, neurotoxic, blockade of the neuromuscular transmission, Similar to anatoxin-a.	Causes potent neuromuscular blockade, mimics neurotransmitter acetylcholine.	200-250 µg/kg b.w. ip in mice.	*Phormidium* *Oscillatoria Aphanizomenon*	[34]
Saxitoxins (STXs) (Neurotoxins)	Nervous system.	Carbamate alkaloids, neurotoxic, causes respiratory distress, myalgia, and muscular paralysis.	Cause paralysis and blocks voltage-gated Na+ channels.	10-30 μg/kg b.w. ip in mice.263µg/kg b.w. orally in mice.	*Anabaena Planktothrix* *Raphidiopsis* *Aphanizomenon* *Lyngbya* *Oxynema*	[13], [20], [37], [38], [39]
β-N-methylamino-L-alanine (BMAA) (Neurotoxins)	Brain, liver, and kidney.	Nonproteinogenic amino acids. Neurotoxic, promote oxidative stress, increase ER stress markers, enhance motor neuron death, and have naturally-occurring isomers, and misfolding and proteotoxic stress.	Overstimulation of NMDA and mGluR5 receptors in CNS and induces oxidative stress, causing neurodegenerative diseases (ALS), Parkinson's disease and Alzheimer’s disease.	300 mg/kg b.w. ip in rats.	*Aphanizomenon, Cylindrospermopsis, Dolichospermum, Planktothrix Microcystis, Nodularia, Synechococcus, Synechocystis*	[40], [41], [42]
L-2,4-diaminobutyric acid (2,4-DAB) (Neurotoxins)	Brain, and liver.	Neurotoxic, an isomer of BMAA, non-protein amino acid, promotes membrane depolarization and causes neurodegenerative disorders, and irreversible damage to fibrosarcoma, glioma, and hepatoma cells.	Neurotoxicity via excitotoxicity mechanisms. hyperirritability, paraparesis with upper extremity tremors.	Not determined.	*Aphanizomenon* *Synechococcus*	[43], [44]
N-(2-aminoethyl) glycine (AEG) (Neurotoxins)	Nervous system.	A naturally occurring isomer of BMAA.	Not fully established.	Not determined.	*Spirulina*	[45]
Lyngbyatoxin (LTX) (Dermatotoxins)	Skin, small intestine, lungs, digestive organs.	Dermatotoxins, indole alkaloids, an inflammatory agent, cause dermatitis, and cellular proliferation, linked with cancer risk.	Activation of protein kinase C (PKC), tumor-promoting activity.	250 μg/kg b.w. in mice.	*Lyngbya,* *Schizotrix, Oscillatoria*	[34], [46], [47]
Aplysiatoxin (APX) (Dermatotoxins)	Skin	Dermatotoxic, phenolic bislactones, promote allergic reactions, tumor, and have anti-proliferative activity, antiviral activity, antileukemia activity, and pro-inflammatory actions.	Protein kinase C activators.	107- 300 μg/kg b.w. ip in mice.	*Lyngbya,* *Schizotrix, Oscillatoria*	[13], [38], [48], [49]
Debromo-aplysiatoxin (Dermatotoxins)	Skin	Phenolic bislactones, a modified version of aplysiatoxin. Dermal toxins, inflammatory agents, cause blisters and necrosis in mammals.	Inflammatory toxins operate through mechanisms similar to those of phorbol esters.	107–117 μg/kg b.w. ip in mice.	*Lyngbya,* *Schizotrix, Oscillatoria*	[13], [38], [48]
Lipo-polysaccharides (LPS) (Irritating Toxins)	Skin and mucosa.	Inflammatory agents, cause dermatitis, blisters, and necrosis in mammals, characteristic of gram-negative bacteria. Promote gastrointestinal illness, allergy, respiratory disease, headache, and fever.	Inflammatory agents, affect any exposed tissue.	40,000–190,000 μg/kg b.w. ip in mice.	All cyanobacteria	[13], [48]

Abbreviations: MC-LR: Microcystin-Leucine Arginine; PP1: Protein phosphatase-1; PP2A: Protein phosphatase 2A; MC: Microcystin; NODs: Nodularins; ATXs: Anatoxin-a; CYNs: Cyclindrospermopsin; STXs: Saxitoxin; WHO: World Health Organization; LPS: Lipopolysaccharides; BMAA: β-*N*-Methylamino-l-alanine; HomoATXa: Homoanatoxin-a; LTX: Lyngbyatoxin; ALS: Amyotrophic lateral sclerosis (ALS); APX: Aplysiatoxin; ip: intra-peritoneal; b.w: Body weight; LD_50_: LD50 lethal dose for 50 % of test animals; ER: Endoplasmic reticulum; PKC: Protein kinase C; NMDA: N-methyl-D-aspartate; AChE: Acetylcholinesterase; AEG: N-(2-aminoethyl) glycine; 2,4-DAB: L-2,4-diaminobutyric acid; CNS: Central Nervous System; mGluR5: Metabotropic glutamate receptor 5; ROS: Reactive oxygen Species.

## 3 Cyanotoxins promoting carcinogenesis

Cancer affects every aspect of the body's physiology by gradually reversing immune, neuroendocrine, metabolic, and possibly microbiological functions. The immune system suppresses the growth of cancer. We call this procedure immunosurveillance. For tumors to progress to disease, altered cells must evade the anticancer immune response [50]. Cyanobacteria are present worldwide, and certain kinds of cyanobacteria produce cyanotoxins that encourage tumor growth. Humans can become exposed to cyanobacteria and cyanotoxins by consuming contaminated food and drinking water. According to previously published research, there appears to be a substantial correlation between the expression of tumor-specific genes involved in Peroxisome Proliferator-Activated Receptors (PPAR) signaling and lipid metabolism and cyanotoxin levels [51]. Primary liver cancer ranks third in terms of cancer-related mortality and is among the sixth most prevalent cancer. MC-LR, first identified as a strong promoter of liver cancer in 1992, was also linked to several additional genes identified in the following 30 years [52]. Several *in vivo* and *in vitro* investigations have shown that certain cyanotoxin, which may have tumor-promoting and perhaps carcinogenic effects, targets the liver and cause tumor growth [22].

### 3.1 Role of MCs in the development and promotion of cancer

According to published research, exposure to MC-LR may be the cause and mechanism of several malignancies that affect both people and animals [26]. Through direct contact with DNA or by generation of reactive oxygen species (ROS), microcystin-LR (MC-LR) acts as a tumor initiator [23]. Chronic exposure to low concentrations of MC-LR has been linked to an increased risk of cancer development. Epidemiological studies conducted in some areas of China have also revealed that MC may be a risk factor for the high incidence of primary liver cancer (PLC) [23].

#### 3.1.1 Mechanism of carcinogenesis

##### 3.1.1.1 MC-LR induces oxidative stress and DNA damage

According to published scientific literature, exposure to MC-LR may increase the production of reactive oxygen species (ROS) and their accumulation in human and animal liver hepatocytes. ROS are primarily responsible for oxidative stress and DNA damage, which trigger the development of liver cancers [24]. Scientific experiments have demonstrated that persistent exposure to MC-LR typically induces ROS production, oxidative DNA damage, and the development of enzymes specialized for oxidative DNA damage [52]. Together, these elements have an impact that starts the carcinogenic process.

##### 3.1.1.2 MC-LR induces DNA methylation

DNA methylation is the best-known and most thoroughly studied epigenetic mechanism and provides a basis for the maintenance of stable phenotypes and switching of gene activities [53]. DNA methylation has also been categorized as MC-LR-induced carcinogenesis in published articles [52], [54], [55]. Alterations in tumor suppressor genes, such as changes in promoter methylation and histone modification patterns, are crucial for the initiation and progression of various cancers, including liver cancer. Nearly all tumors display abnormal DNA methylation in specific genes, suggesting that this modification can serve as a molecular marker for cancer [55]. It has been suggested that differential methylation of CpG sites in the coding sequences of genes and the promoter occurs during MC-LR exposure, which leads to differential gene expression; in this way, MC-LR plays a role in cancer formation, invasion, and migration [52].

##### 3.1.1.3 MC-LR-dependent inhibition of protein phosphatases 2 A in hepatocytes

Protein phosphatase 2 A (PP2A) is a significant serine-threonine phosphatase in nearly all human cells. Its primary function is to maintain cell homeostasis by inhibiting intracellular signaling pathways dependent on kinases. Both the activation of oncogenic kinases and the suppression of tumor suppressors are necessary for the development of cancer. Given that PP2A has been discovered to be both functionally inactivated and genetically altered in a large number of solid tumors, experimental data suggest that PP2A is, in fact, an authentic tumor suppressor [56]. Published research has demonstrated that MC-LR inhibits protein phosphatase 2A (PP2A), which functions as a tumor suppressor by interfering with differentiation, proliferation, apoptosis, and DNA repair pathways. As a result, PP2A inhibition induced by MC-LR causes hyperphosphorylation of cytokeratins 8 and 18, which results in aberrant proliferation of hepatocytes and thus promotes the development of cancer [52].

##### 3.1.1.4 MC-LR regulates expression of TGF-β1 and CST3

In a recent study, MC-LR has been linked to the development and promotion of colorectal cancer (CRC). However, the specific mechanism by which MC-LR influences CRC progression within the tumor microenvironment (TME) remains unclear. The findings showed that MC-LR enhances CRC cell migration by increasing the expression and secretion of transforming growth factor-beta 1 (TGF-β1) in M2 macrophages while suppressing cystatin C (CST3) expression in CRC cells. Neutralizing TGF-β1 restored CST3 levels in CRC cells while overexpressing CST3 reduced TGF-β1 expression in M2 macrophages, both impairing the MC-LR-induced cell migration in the co-culture model. Hence, it was revealed that MC-LR promotes CRC cell migration by increasing TGF-β1 expression in M2 macrophages, which in turn suppresses CST3 in CRC cells [35].

##### 3.1.1.5 MC-LR-induced CXCL1/IGHG1 signalling pathway

In a recent study, co-culture models and xenograft mouse experiments confirmed that MC-LR stimulates tumor-associated macrophages (TAMs) to secrete CXCL1, which in turns promotes colorectal cancer (CRC) cell proliferation, migration, and invasion. Additionally, immunoprecipitation-mass spectrometry (IP-MS) revealed that TAM-derived CXCL1 interacts with immunoglobulin heavy constant gamma 1 (IGHG1) in CRC cells, and enhances cancer cell proliferation and migration. Silencing IGHG1 reduces these effects, thus highlighting its role in CRC progression. Molecular docking, co-immunoprecipitation, and immunofluorescence further validated the CXCL1-IGHG1 interaction [36].

##### 3.1.1.6 MC-LR induced matrix metalloproteinase up-regulation

A recent study suggests that MC-LR activate the PI3K/AKT pathway, which leads to increased matrix metalloproteinase-13 (MMP-13) expression, resulting in enhanced migration and invasion of Duke's Lymphoma Disease 1 (DLD-1) and Human Tumor 29 (HT-29) cells. MC-LR treatment enhance the migration and invasion of DLD-1, HT-29 cells, which correlated with increased messenger RNA (mRNA) and protein levels of MMP-13 [57]. Other studies also indicated that chronic exposure to MC-LR could alter metalloproteinase-2/-9 (MMP-2/-9) expressions and stimulate cancer cell migration [27], [58].

##### 3.1.1.7 MC-LR induced inflammatory responses

A study suggested that even at low concentrations, MC-LR can lead to excessive ROS production in HepG2 cells. Furthermore, prolonged exposure to low concentrations of MC-LR significantly increased the expression of nuclear factor kappa-light-chain-enhancer of activated B cells (NF-κB) p65, cyclooxygenase-2 (COX-2), inducible nitric oxide synthase (iNOS), tumor necrosis factor-alpha (TNF-α), interleukin-1 beta (IL-1β), and interleukin-6 (IL-6) in the cells. This indicates that long-term low-level MC-LR exposure can trigger inflammatory responses in HepG2 cells, potentially contributing to MC-induced human hepatitis and hepatocarcinoma [59].

##### 3.1.1.8 MC-LR induced regulation of HOXB4

In a study MC-LR was confirmed to enhance HOXB4 expression, promoting the proliferation and migration of colon adenocarcinoma (Caco2) cells. MC-LR may accelerate CRC progression by increasing C-myc levels, which in turn elevates HOXB4 expression and enhances the proportion of cells in the S phase of the cell cycle, thereby boosting Caco2 cell proliferation [60].

##### 3.1.1.9 MC-LR induced expression of cadherin-11

The results showed that microcystin-LR activated the expression of cadherin 11 and increased cell migration and invasion in HT-29 cells. To further examine the relationship between microcystin-LR-induced upregulation of cadherin 11 and the increased motility and invasiveness of HT-29 cells, the researchers conducted a knockdown of cadherin 11 using small interfering RNA in HT-29 cells. Subsequent Transwell assays confirmed that the enhancement of migration and invasion induced by microcystin-LR was significantly reduced following the knockdown of cadherin 11 using cadherin 11 small interfering RNA in HT-29 cells. These findings suggest that microcystin-LR may function as a cadherin 11 activator, promoting the migration and invasiveness of HT-29 cells [61].

##### 3.1.1.10 MC-LR and IRE1α/XBP1 signalling pathway

**Fig. 1:**
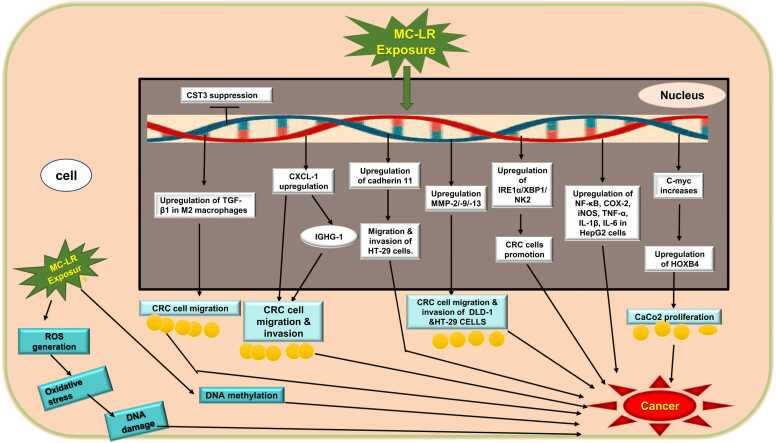
Shows molecular mechanisms by which MC-LR induces cancer development and promotion. Exposure to MC-LR leads to ROS generation, oxidative stress and DNA damage as well as DNA methylation. It also up regulates the expression of TGF-β1 in M2 macrophages, CXCL-1, IGHG-1, cadherin 11, MMP-2/-9/-13, IRE1α/XBP1/NK2, NF-κB, COX-2, iNOS, TNF-α, IL-1β, IL-6 and HOXB4 genes within the cell. These molecular mechanisms all together lead to cancer development and promotion. Abbreviations: CST3: Cystatin C; TGF-β1: Transforming Growth Factor Beta 1; IGHG-1: Immunoglobulin Heavy Constant Gamma 1; HT-29: human colorectal adenocarcinoma; MMP-2/-9/-13: Matrix Metalloproteinase-2/-9/-13; CRC: Colorectal Cancer; NF-κB: Nuclear Factor kappa-light-chain-enhancer of activated B cells; COX-2: Cyclooxygenase-2; iNOS: Inducible Nitric Oxide Synthase; TNF-α: Tumor Necrosis Factor Alpha; IL-1β: Interleukin-1 Beta; IL-6: Interleukin-6; HOXB4: Homeobox B4.

A study in a mouse model revealed that MC-LR exposure resulted in the progression from adenoma to adenocarcinoma by increased macrophage infiltration. The study found that MC-LR exposure activated the inositol-requiring enzyme 1 alpha (IRE1α)/X-box binding protein 1 (XBP1) pathway in CRC cells, which influenced the polarization of macrophages toward the M2 phenotype in co-culture experiments. Additionally, hexokinase 2 (HK2), a downstream target of the IRE1α/XBP1 pathway, was identified as a key regulator of glycolysis and lactate production. The IRE1α/XBP1/HK2 axis promoted lactate production in CRC cells, which further enhanced M2 macrophage polarization. Co-culture experiments with MC-LR-exposed CRC cells and macrophages showed that inhibiting the IRE1α/XBP1 pathway with 4μ8 C and blocking hexokinase activity with 2-deoxyglucose (2-DG) suppressed M2 macrophage-induced CRC cell migration, colony formation, and polarization [62]. (Fig. 1)

### 3.2 Potential indirect roles of anatoxin-a in the development and promotion of cancer

Anatoxin-a is a cyanotoxin detected worldwide. Neurotoxic activity is generally observed during exposure to anatoxin-a. To our knowledge, Sieroslawska, A., & Rymuszka, A. (2010) first reported the genotoxic effects of anatoxin-a, and the obtained results show, that both MC-LR and anatoxin-a possess genotoxic potential with anatoxin-a being active at an even lower concentration, than MC-LR [30]. However, there is no direct evidence that anatoxin-a causes cancer [63], but according to this genotoxic study, exposure to toxins may cause cytotoxicity, which is characterized by DNA breakage, loss of viability, and lactate dehydrogenase leakage in addition to mitochondrial dysfunction. It is also responsible for caspase activation and the production of reactive oxygen species [30]. The carcinogenic potential and mechanism of anatoxin-a in people and animals is mostly unknown. Anatoxin-a is supposed to be carcinogenic because it causes inflammation, and oxidative stress, and possibly disrupts certain cell signaling pathways. Nevertheless, there is no concrete evidence that anatoxin-a causes cancer. Anatoxin-a uptake has been shown to disrupt oxidative stress in aquatic plants in an experimental investigation. The degree and length of exposure determine the occurrence of oxidative damage in plant cells [63]. A recent study reported that Anatoxin-a (ATX-a) did not show any mutagenic effects alone or when combined with cylindrospermopsin (CYN) in the Ames test. However, both ATX-a and its mixture with CYN caused genotoxic effects in the L5178Y Tk+/− cell line during the in vitro micronucleus (MN) assay. ATX-a only showed genotoxicity when the S9 metabolic mix was absent. Despite this, the ATX-a/CYN mixture shows concentration-dependent genotoxicity, but only when the S9 mix was present. This study highlights the importance of studying the combined effects of cyanotoxins since mixtures can behave differently from individual toxins which provides a more accurate understanding of their effects [64].

### 3.3 Role of nodularin in the development and promotion of cancer

Nodularin is primarily associated with hepatocellular carcinoma (HCC). The main organ that nodularins (NODs) target is the liver, and the main mechanism of their toxicity is the suppression of eukaryotic protein serine or threonine phosphatases 1, 2A, and 3. Disruption of the hepatic architecture caused by the loss of cell-cell adhesion, cytoskeleton alteration and rearrangement, and disruption of numerous cellular processes, results in hyper-phosphorylation of certain cellular proteins, intrahepatic hemorrhage, and hepatic insufficiency [65]. These toxins when entering cells cause oxidative stress, lipid peroxidation, proteins, and DNA damage. They achieve this within cells by generating reactive oxygen species [39]. Most people refer to NODs as tumor promoters. Their greater tumor-promoting effect than that of MCs is attributed to their inhibition of protein phosphatase activity. They can initiate tumors and are genotoxic *in vitro*, according to scientific research [65].

#### 3.3.1 Nodularin mechanism of carcinogenesis

Nodularin promotes cancer by causing oxidative DNA damage, disrupting chromosome segregation, and interfering with essential enzymes. It allows damaged cells to survive by blocking apoptosis and induces gene instability, all of which contribute to the progression of tumors, especially in the liver [65].

##### 3.3.1.1 Tumor initiation and tumor promotion

Nodularin can increase tumor necrosis factor-α (TNF-α) gene expression. TNF-α is known to promote tumor development by inducing inflammation and creating a microenvironment that supports cancer growth. Nodularin also activates several early-response genes like c-jun, jun B, jun D, c-fos, fos B, and fra-1, which regulate cell survival and division. This prolonged gene expression contributes to uncontrolled cell growth and thus development of cancer [39]. These investigations support that nodularin may also function as a tumor initiator.

##### 3.3.1.2 Inhibition of protein phosphatase (PPI)

The mechanism of nodularin that is often proposed is its inhibition of protein phosphatases. PPIs cause the phosphorylation of some biological proteins to increase. These results in the hyperphosphorylation of the cell's intermediate filaments, cytokeratins 8 and 18, which alter the morphology of the entire cell due to cytoskeletal rearrangements [66]. Protein phosphatase (PPI) inhibition causes cellular dysregulation, which might eventually result in cancer.

##### 3.3.1.3 Oxidative stress and DNA damage

Nodularin typically produces intracellular ROS, such as superoxide and hydroxyl radicals. These radicals cause oxidative stress in cells, which in turn damages proteins, lipid peroxidation, and DNA [58]. Mutations caused by oxidative stress and DNA damage are the main causes of cancer in both humans and animals. Research has demonstrated through experimentation that nodularin induces oxidative stress. Using qPCR, it was possible to identify variations in the expression of p53 pathway genes related to oxidative stress, apoptosis, and DNA damage. ROS are produced at noncytotoxic concentrations, which in turn lead to an increase in oxidative DNA damage within the cell [67].

##### 3.3.1.4 Induction of aneuploidy (Chromosomal imbalance)

Nodularin can cause abnormal chromosome separation during cell division, leading to aneuploidy. Using micronucleus assays, the researchers found that nodularin-treated cells often had centromere-positive micronuclei, meaning whole chromosomes failed to properly segregate during division. This chromosomal imbalance contributes to cancer development by disrupting normal cellular regulation [68].

##### 3.3.1.5 Inducing apoptosis

While nodularin can trigger apoptosis (programmed cell death) in some cells, it can also leave others damaged but alive, creating the conditions for cancer. The study found that at specific doses and over time, nodularin caused early-stage and late-stage apoptosis, as well as necrosis (uncontrolled cell death). If damaged cells survive and evade apoptosis, they may continue to divide abnormally and contribute to cancer development [68].

##### 3.3.1.6 Modulation of gene expression related to DNA repair and apoptosis

Nodularin affects the expression of certain genes involved in DNA repair and cell death. It upregulates the B-cell lymphoma 2 (BCL2) gene, which helps cells resist apoptosis (programmed cell death), allowing damaged cells to survive and proliferate. At the same time, it alters the expression of DNA-damage response genes, such as growth arrest and DNA damage-inducible α (GADD45α), showing that the cells are trying to repair the damage [65].

### 3.4 Role of cylindrospermopsin in the development and promotion of cancer

The polyketide-derived alkaloid CYN was initially identified in 1992 in the cyanobacterium *Raphidiopsis raciborskii* [69]. Even though other organs may also be impacted, the liver is the main target, indicating the cytotoxic qualities of CYN. It is primarily responsible for HCC in the liver [70]. In experimental animals, CYN exposure generates fatty liver and hepatonecrosis, with extrahepatic lesions of varying severity and location [71]. The harmful effects of CYN were shown to cause harm to cell organelles and reduce cell viability. In addition to the liver, other organs affected by CYN include the heart, lung, kidney, ovary, spleen, T cells, neutrophils, and vascular endothelium [72]. Because CYN suppresses apoptosis and increases oxidative stress, it can be suggested that it is carcinogenic and may play a role in the initiation and progression of tumors.

#### 3.4.1 Mechanism of carcinogenesis

A potentially harmful mechanism of CYN is the irreversible suppression of protein and glutathione (GSH) production. Numerous investigations have indicated that cytochrome p450 metabolizes CYN and that it exhibits both cytotoxic and genotoxic properties, suggesting that CYN is pro-genotoxic. Research has indicated that in HepG2 cells, CYN and its combination with MC-LR caused DNA double-strand breaks and thus induced genomic instability [70]. Evidence for the role of glutathione conjugation and cytochrome P-450 in CYN metabolism has been found both *in vitro* and *in vivo*. When the sublethal dose of CYN (70 μg kg−1) is injected intratracheally, mice exhibit oxidative stress, labored breathing, and inflammation of the pulmonary parenchyma [73]. CYN inhibited both glutathione synthesis and protein synthesis in a primary hepatocyte culture [71]. CYN inhibits both the translation of messenger RNA and the expression of genes involved in ribosome synthesis. Additionally, it prevents rat hepatocyte primary cultures from synthesizing glutathione. It has also been demonstrated that it causes oxidative stress by lowering the production of glutathione. The suppression of protein synthesis, oxidative stress, genotoxicity, and histopathological changes are the causes of neurotoxicity caused by CYN [72]. Taken together, these data indicate that CYN is carcinogenic and may be the starting point for the development of tumors through the induction of oxidative stress, inhibition of protein synthesis, DNA damage, and genotoxicity. A study investigated the cytotoxic effects of MC-LR and CYN, individually and in combination, on the SH-SY5Y human neuroblastoma cell line. Both toxins showed cytotoxic effects in both differentiated and undifferentiated cells. CYN was found to be more toxic than MC-LR. In combination, the toxins exhibited greater cytotoxicity in undifferentiated cells compared to individual exposure, but in differentiated cells, their combined effect was similar to that of MC-LR alone. Morphological observations revealed apoptotic processes at all tested concentrations, indicating cellular damage [74]. CYN is Recognized for its genotoxicity and potential carcinogenic. A study examined whether non-cytotoxic concentrations of CYN cause DNA damage through oxidative stress and whether it triggers apoptosis in HepG2 liver cells. Significant increases in ROS levels, with steadily rose with incubation time were observed. After 12 and 24 hours of exposure, CYN significantly increased DNA strand breaks, though without oxidative DNA damage, indicating that oxidative stress plays a minor role in CYN-induced genotoxicity. It is revealed that CYN-induced DNA damage is not linked to oxidative stress or apoptosis. Importantly, the lack of apoptosis means damaged cells are not removed, increasing the risk of mutations and potentially contributing to carcinogenesis [75].

A study investigated the genotoxic effects of CYN on HepG2 cells and found that prolonged exposure to CYN (72 hours) induces DNA double-strand breaks. Flow cytometry analysis showed that CYN disrupted the cell cycle, inducing G0/G1 phase arrest after 24 hours and S-phase arrest after 72–96 hours of exposure. These findings provide new evidence that CYN acts as a direct genotoxin, causing DNA double-strand breaks [76]. In another study CYN also altered the expression of the P53 gene and its associated DNA damage response genes, including MDM2 and GADD45α, as well as BCL-2 and BAX, which are linked to apoptosis. Furthermore, CYN affected oxidative stress-related genes such as GPX1, SOD1, GSR, and GCLC, though no changes were observed in CDKN1A or CAT expression.

These findings confirm that CYN is genotoxic and that lymphocytes are potential targets of CYN-induced genetic damage, emphasizing the toxin's potential health risks [77]. A study analyzed the cellular responses of HepG2 liver cells exposed to a non-cytotoxic but genotoxic dose of CYN (0.5 micrograms per milliliter) over 12 and 24 hours. The results showed increased expression of immediate-early response genes, such as those from the FOS and JUN families, and suggested significant involvement of P53 and nuclear factor kappa-light-chain-enhancer of activated B cells (NF-κB) pathways. There was also a notable up-regulation of several key stress response genes, such as GADD45A, GADD45B, cyclin-dependent kinase inhibitors (CDKN1A and CDKN2B) and checkpoint kinase 1 (CHEK1) [78]. Taken together, these data indicate that CYN is carcinogenic and may be the starting point for the development of tumors through the induction of oxidative stress, inhibition of protein synthesis, DNA damage, and genotoxicity.

### 3.5 Potential indirect roles of saxitoxin in the development and promotion of cancer

**Fig. 2:**
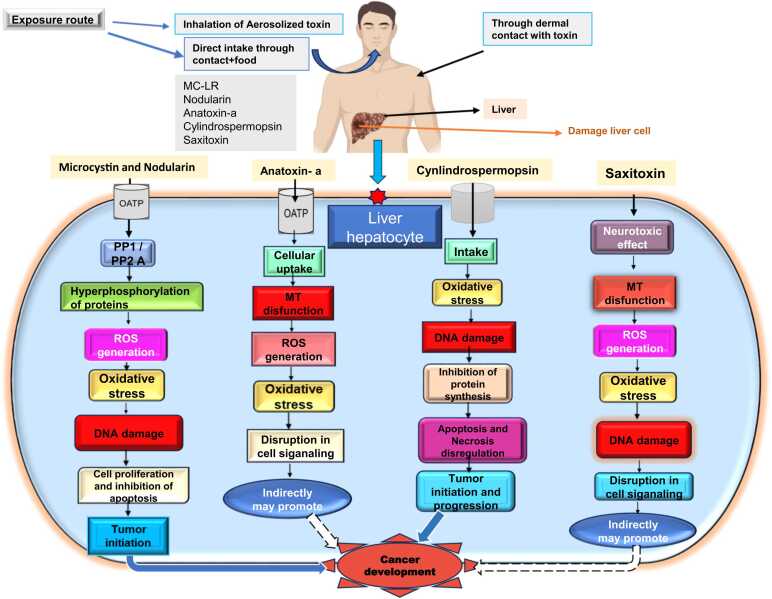
Shows the possible exposure route of cyanotoxins to animals and their uptake in liver hepatocytes, leading to carcinogenesis. MC-LR and nodularin inhibit the protein phosphatases, leading to hyperphosphorylation of proteins and generation of ROS, hence causing oxidative stress and DNA damage in the cells, leading to cell proliferation and inhibition of apoptosis. Cylindrospermopsin inhibits protein synthesis, leading to apoptosis and necrosis dysregulation, and contributing to tumor initiation and progression. Anatoxin-a and Saxitoxin generate ROS and lead to oxidative stress within the cells, thus disturbing cell signaling. By utilizing these pathways different cyanotoxins play their role in the development and promotion of cancers. Abbreviations: OATP: Organic Anion Transporting Polypeptide; PP1/PP2A: Protein Phosphatase/ Protein Phosphatase 2A; ROS: Reactive oxygen species**;** MT: Mitochondria.

When saxitoxins are present in large concentrations, they can cause paralysis and even death by obstructing voltage-gated sodium channels. Other neuronal channels, including calcium and potassium channels, can also be obstructed by saxitoxin. Long-term exposure to saxitoxin typically results in detrimental health consequences for antioxidant systems and DNA damage, as shown in mammalian models [79]. According to previous studies; STXs may bioaccumulate via the food chain and cause fish and other aquatic species, including livestock, to perish [80]. Saxitoxin binds to voltage-gated sodium channels to inhibit sodium ion inflow in nerve and muscle cells. This can disrupt normal cell signaling pathways, change cellular development and apoptosis, and ultimately may contribute to tumors. Saxitoxin binds specifically to the action potential Na+ ionophore in neuroblastoma cells. [81]. Research has also indicated that saxitoxin may cause cells to produce reactive oxygen species, which are mainly responsible for lipid, protein, and DNA damage [79]. Although saxitoxins (STXs) are primarily recognized for their neurotoxic effects, they may have carcinogenic qualities but data on STX-mediated cytotoxic and genotoxic effects are still scare and there is no direct evidence that links saxitoxin to cancer [82]. A recent study evaluated the cytotoxicity and chromosome instability induced by saxitoxin (STX) in the SHSY-5Y human cell line. The cytokinesis-block micronucleus (CBMN) assay showed that STX concentrations between 2.5 and 10 micrograms per liter (µg/L) induced cytostasis and chromosome instability in a dose-dependent manner. Apoptosis was observed after exposure to 10 µg/L of STX. This study revealed that STX also interacts with proteins involved in the acetylcholine pathway, cell cycle regulation, and apoptosis and it proposed that STX induces cytotoxic and mutagenic effects in SHSY-5Y cells [83]. In a second study nine proteins were found to be altered due to saxitoxin exposure including 14–3–3 beta (1433B), alpha enolase (ENO1), and cofilin 2 (CFL2). These identified proteins are involved in key cellular processes, including apoptosis, cytoskeletal maintenance, membrane potential regulation, and mitochondrial function. These changes suggest that even the low-dose exposure to saxitoxin can impact these pathways, which aligns with previous reports of saxitoxin-induced genotoxicity and neurotoxicity [84]. The diagram below illustrates how various cyanotoxins cause cancer (Fig. 2).

## 4 Mitigation strategies for cyanotoxins and health protection approaches

Cyanobacteria blooms or harmful algal blooms (HABs), release different cyanotoxins into natural water bodies and constitute a serious risk to health and the environment. A combination of strategies is used to reduce these risks to ensure that water is safe for drinking and recreational activities [85]**.**

### 4.1 Nutrient control

Excess nutrients, especially nitrogen and phosphorus, drive cyanobacteria growth. Limiting fertilizers and sewage inflow into lakes can slow bloom development [85], [86]. Both nitrogen and phosphorus need to be managed since many cyanobacteria thrive on both. Reducing only one may not be effective, and dual management is critical for sustainable control [86], [87].

### 4.2 Chemical treatments

Copper-based chemicals effectively kill cyanobacteria but can harm other aquatic life. Hydrogen peroxide is a safer alternative, breaking down cyanobacteria without significant environmental harm. However, both methods are more suited for smaller water bodies [85], [86], [87]. Phosphorus precipitation (Phoslock) is also useful in which phosphorus binds in sediments to prevent it from being available for cyanobacteria growth, helping limit future blooms [86]. Chemicals like potassium permanganate, chlorine, and ozone can also break down these toxins. Additionally, advanced oxidation processes (AOPs), such as UV/ozone combinations, are highly effective at degrading cyanotoxins but are expensive and need careful monitoring to avoid harmful by-products. It is mostly practical for smaller-scale applications, such as drinking water treatment plants [88], [89].

### 4.3 Biological control

Introducing fish or zooplankton that feed on cyanobacteria can help reduce blooms. Care is needed to ensure this approach does not unintentionally encourage toxic species that are harder to control. Some bacteria and viruses naturally attack cyanobacteria, but this strategy is still under research for safety and effectiveness [85], [86], [87]. Aquatic plants that compete for nutrients with cyanobacteria can reduce bloom growth. Some plants release natural chemicals (allelochemicals) that inhibit cyanobacteria growth [89].

### 4.4 Physical and mechanical controls

Increasing water movement through aeration or flushing helps disrupt the conditions cyanobacteria need to grow, such as warm and stagnant waters. Removing or capping nutrient-rich sediments can prevent internal nutrient release, reducing the likelihood of future blooms [86]. Techniques such as ultrafiltration and reverse osmosis are also used to remove cyanobacteria and dissolved toxins. However, these methods are costly and prone to fouling, requiring frequent maintenance [88]. Adding chemicals like aluminium salts helps clump cyanobacteria cells, making it easier to filter them out. However, breaking cells can release toxins, which must be removed later through additional processes [88], [89].

### 4.5 Nanoparticles as a control method

Nanoparticles (NPs) can disrupt cyanobacteria by damaging cell membranes, reducing buoyancy, and interfering with light absorption [90]. Some NPs generate ROS, which degrade toxins and kill cyanobacteria. Metallic NPs (like titanium dioxide and zinc oxide) break down toxins through photocatalysis when exposed to light. Carbon-based NPs (like graphene) inhibit cyanobacteria growth by blocking light and interacting with cell surfaces. NPs like titanium dioxide (TiO_2_) use light energy to degrade cyanotoxins such as microcystins. This process prevents toxin buildup in water supplies. Magnetic NPs offer a way to collect and remove cyanobacteria quickly by attracting them with magnets [90].

### 4.6 Health protection measures

**Fig. 3:**
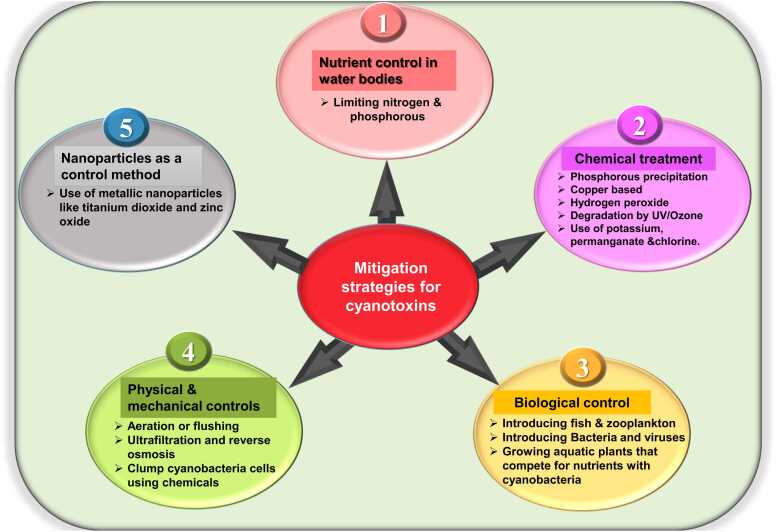
Shows the different strategies to mitigate cyanotoxins including nutrient control in water bodies and chemical, biological, physical and mechanical control along with the use of nanoparticles.

Regular monitoring with real-time testing tools helps detect cyanotoxins early, ensuring quick responses to protect public health. Following health advisories from authorities like the WHO is also crucial for ensuring safe recreational and drinking water use [85], [86]. Health advisories guide the public on safe water usage during bloom events, helping protect against exposure through drinking and recreational activities [88]. Advanced treatment methods like filtration, activated carbon, and oxidation processes (e.g., ozonation) are effective at removing toxins from drinking water [89]. During bloom events, switching to alternative sources of water or using bottled water can reduce the risk of cyanotoxin exposure. In areas prone to cyanobacterial blooms, informing the public about the dangers of using contaminated water for drinking, cooking, and recreational activities is vital to preventing acute poisoning incidents [91]. The diagram below illustrates different mitigation strategies for cyanotoxins (Fig. 3).

## 5 Conclusion

Cyanotoxins are generally produced by different species of cyanobacteria in aquatic ecosystems, and their exposure to humans and other animals is primarily responsible for numerous health issues, including hepatotoxicity, neurotoxicity, and dermatotoxicity. This review highlights that several cyanotoxins, such as microcystins, nodularins, and cylindrospermopsin, can contribute directly to cancer development through a variety of mechanisms. Review findings indicate that these cyanotoxin has carcinogenic properties and may promote cancer development**.** Mechanism-based studies have revealed that these cyanotoxins can induce oxidative stress and DNA damage as well as disrupt cellular signaling pathways in normal cells and contribute to carcinogenesis by acting as potential carcinogens. Microcystin and nodularin inhibit protein phosphatases (PP1 and PP2A), which results in uncontrolled cell proliferation and thus tumor promotion. Cylindrospermopsin inhibits protein synthesis and has genotoxic effects and thus may initiate tumor formation. Anatoxin-a and saxitoxin mainly have neurotoxic effects, but they may play an indirect role in cancer development by inducing oxidative stress and DNA damage in cells hence, further studies are needed to elucidate their role in the promotion and development of cancer. Mitigating the effects associated with the exposure of cyanotoxins to animals and humans requires a combined strategy, such as nutrient control to water bodies and chemical, biological, physical, as well as mechanical treatments of infected water bodies along with some public health protection measures.

## CRediT authorship contribution statement

**Roshni Rajpoot:** Data curation, Formal analysis, Writing – review & editing. **Nidhi Gupta:** Writing – original draft, Investigation, Formal analysis, Data curation. **Chandrama Prakash Upadhyaya:** Writing – review & editing, Project administration. **Mohammed Latif Khan:** Writing – review & editing, Project administration. **Raj Kumar Koiri:** Writing – review & editing, Supervision, Project administration, Funding acquisition, Conceptualization. **Siddharth Rajput:** Writing – original draft, Methodology, Investigation, Formal analysis, Conceptualization. **Shruti Jain:** Writing – original draft, Methodology, Formal analysis, Data curation, Conceptualization. **Debabrata Dash:** Investigation, Formal analysis, Data curation.

## Declaration of Competing Interest

The authors declare the following financial interests/personal relationships which may be considered as potential competing interests, Raj Kumar Koiri reports financial support was provided by Science and Engineering Research Board. Raj Kumar Koiri reports financial support was provided by India Ministry of Science & Technology Department of Science and Technology. Raj Kumar Koiri reports financial support was provided by India Ministry of Science & Technology Department of Biotechnology. Debabrata Dash reports financial support was provided by Indian Council of Medical Research. If there are other authors, they declare that they have no known competing financial interests or personal relationships that could have appeared to influence the work reported in this paper.

## Data Availability

No data was used for the research described in the article.
